# Gene Editing and Modulation: the Holy Grail for the Genetic Epilepsies?

**DOI:** 10.1007/s13311-021-01081-y

**Published:** 2021-07-07

**Authors:** Jenna C. Carpenter, Gabriele Lignani

**Affiliations:** Department of Clinical and Experimental Epilepsy, UCL Queen Square Institute of Neurology, Queen Square House, London, WC1N 3BG UK

**Keywords:** Gene editing, Epilepsy, Channelopathies, CRISPR, Development

## Abstract

Epilepsy is a complex neurological disorder for which there are a large number of monogenic subtypes. Monogenic epilepsies are often severe and disabling, featuring drug-resistant seizures and significant developmental comorbidities. These disorders are potentially amenable to a precision medicine approach, of which genome editing using CRISPR/Cas represents the holy grail. Here we consider mutations in some of the most ‘common’ rare epilepsy genes and discuss the different CRISPR/Cas approaches that could be taken to cure these disorders. We consider scenarios where CRISPR-mediated gene modulation could serve as an effective therapeutic strategy and discuss whether a single gene corrective approach could hold therapeutic potential in the context of homeostatic compensation in the developing, highly dynamic brain. Despite an incomplete understanding of the mechanisms of the genetic epilepsies and current limitations of gene editing tools, CRISPR-mediated approaches have game-changing potential in the treatment of genetic epilepsy over the next decade.

**Supplementary Information:**

The online version contains supplementary material available at 10.1007/s13311-021-01081-y.

## Introduction

Epilepsy affects 1% of the population (50 million people worldwide), and 30% of patients are drug-resistant, with not many available options for treatments [1]. Novel antiseizure drugs have had little impact on drug-resistant epilepsies; thus, new treatments are an urgent unmet clinical need [2]. Gene therapy and editing holds promise as a rational treatment for epilepsy. Recent developments in the gene editing field provide new hope for patients, and clinical trials using CRISPR to treat disease have already begun, or are in the pipeline [3, 4]. Here, we discuss potential gene editing strategies that could be game changers in the treatment of genetic epilepsy.

## The Genetic Architecture of Epilepsy

Epilepsy is a complex neurological disorder that displays high heritability, with a genetic contribution estimated in 70–80% of cases [5, 6]. The genetic architecture of epilepsy is contributed to by a large number of individually rare monogenic subtypes, mostly caused by de novo mutations. The more common forms of epilepsy are hypothesised to be caused by a complex interaction of individually rare and more common variants in multiple susceptibility genes, but this remains speculative [5]. Over the past two decades, the genomic revolution has led to the identification of nearly 1000 epilepsy associated genes with Mendelian inheritance (OMIM). Mutations occurring in ion channels account for ~ 25% of monogenic epilepsy [7]. A high percentage of cases are also caused by mutations in genes associated with synaptic transmission, cortical development, and metabolic function [8].

Importantly, an age-dependence of epilepsy incidence has been reported, with the highest likelihood of a molecular diagnosis occurring under the age of 5 years [8]. The majority of diagnoses feature rare, highly deleterious variants concentrated in a small number of recurrently implicated genes (i.e. PRRT2, *SCN1A, KCNQ2, CDKL5, SCN2A, STXBP1*, SLC2A1, and *PCDH19),* with the 10 most represented genes accounting for ~ 80% of cases of monogenic epilepsy [8, 9]. Mutations in these genes often result in developmental and epileptic encephalopathy (DEE), which features frequent seizures, severe neurodevelopmental comorbidities, and drug resistance in > 50% of cases [10]. Collectively, the DEEs are the most common rare monogenic epilepsies (1:2000 births) [5] that contribute the highest proportion of drug resistant epilepsy and worst outcomes and would therefore highly benefit from a targeted gene therapy approach.

How feasible is a targeted gene editing approach for the DEEs? DEEs exhibit a high degree of phenotypic, inter- and intra-genetic heterogeneity, which can make a truly personalised medicine approach challenging. A wide spectrum of de novo mutations in the same gene can cause DEEs, rendering a mutation-specific precision medicine approach complex and financially unsustainable. Furthermore, we should also consider that genotype–phenotype relationships are complex: variable phenotypes can arise from different mutations occurring in the same gene, as exemplified by mutations in *SCN1A*, where the phenotypic spectrum can range from generalised epilepsy with febrile seizures plus (GEFS +) to Dravet syndrome [11]. Such phenotypic pleiotropy, which is contributed to by genetic background and disease modifier genes, is likely to influence therapeutic outcomes following single gene manipulation in different patients.

Despite the heterogeneity of mutations and phenotypes associated with individual genes, there are genetic tools available that could potentially be used to rescue mutations leading either to loss-of-function (LOF), gain-of-function (GOF), or to a dominant negative mode of action [12].

## CRISPR/Cas Genome Editing for Genetic Epilepsy

Since its recent discovery as part of the adaptive immune system of bacteria/archea, the CRISPR/Cas system has been rapidly adapted for genome engineering in mammalian cells [3]. Gene editing is achieved using an RNA-guided, CRISPR-associated (Cas) DNA endonuclease that precisely generates double-strand DNA breaks (DSB) at a target DNA locus [13]. The ‘programmability’ of the Cas protein by the design of a short (20 nucleotide) guide RNA sequence (sgRNA) has opened the possibility to target the full complement of genes in the human genome at a base pair resolution, such that CRISPR/Cas genome editing is now considered the gold-standard in precision medicine.

The naturally occurring, Cas9 endonuclease from the Type II CRISPR system has been the most commonly used for genome editing applications, and multiple engineered Cas9 variants have been developed, with different editing capabilities, specificities, targeting ranges and sizes, to facilitate viral delivery (reviewed in Anzalone et al. [13]). The type and location of the desired edit determine the chosen tool. Common desired edits for the treatment of genetic disorders would include the correction of point mutations (base editing/prime editing); deletion of base pairs (Cas9 endonuclease), insertion of base pairs (Homology-independent-targeted-integration, HITI), or a combination of the above [13].

The type of genomic edit that occurs following the introduction of DNA breaks crucially depends upon endogenous cellular DNA repair pathways, of which there are two branches: homology-dependent repair (HDR) and non-homologous end joining (NHEJ) [14]. HDR uses a DNA template to precisely repair DSBs, whereas NHEJ is an error-prone, template-free repair pathway, in which DNA ends are rapidly re-ligated, often with the stochastic insertion or deletion (indels) of base-pairs at the break-site. NHEJ is the most active repair in mammalian post-mitotic cells, such as neurons [14, 15]. We therefore constrain our discussion below to the editing agents and outcomes that rely on NHEJ.

### Cas9 Endonuclease-Mediated Gene Editing

CRISPR/Cas9 is most commonly used to efficiently and selectively disrupt protein coding exons via indel formation following NHEJ, which can generate frameshifts that lead to premature termination via introduced stop codons [14]. Gene disruption as a therapeutic strategy has yet to be adopted for epilepsy, presumably because most epilepsy genes are under tight regulatory control and knockout would be deleterious in the vast majority of cases. If technology for allele-specific targeting were to be improved [16], however, then NHEJ might be harnessed to destroy a gene variant that acts in a dominant or dominant negative manner, in a context where haploinsufficiency of the gene product results in a less severe phenotype [17]. A possibility for genetic epilepsy, and an as of yet untested strategy, could be to use CRISPR to destroy the splice-site of the recently discovered poison exon (20 N) in *SCN1A*, in order to permanently upregulate *SCN1A* expression in Dravet syndrome [18, 19]. Proof-of-principle of such an approach has been demonstrated using Targeted Augmentation of Nuclear Gene Output (TANGO) technology, which uses an antisense oligonucleotide targeted to the poison exon of *Scn1a* to reduce a non-productive alternative splicing event [20]*.* TANGO was able to increase the level of functional *Scn1a* transcripts and WT Nav1.1 protein by decreasing the level of non-productive mRNA, and is already in Phase I/II clinical trial [20].

If a double-stranded DNA template is provided, however, NHEJ can be harnessed for the insertion of sequences in a process called homology-independent targeted integration (HITI), or terminal microhomology mediated end joining (MMEJ), where microhomology exists between the ends of the template and the genomic target [21]. HITI can be used to reinsert deleted sequences [22], or to insert full coding sequences, blocking the downstream expression of mutated genes [23]. Genetic epilepsies caused by deletions, such as in *NRXN1* [24] and selected cases of *PCDH19* [25], could be potentially treated using these approaches. Such an approach could also be used to bypass the expression of a dominant GOF mutant protein [23].

### Base and Prime Editing

In recent years, the CRISPR toolbox has been expanded with the advent of base editors (reviewed in Rees and Liu [26]). Base editors are generated via the fusion of a Cas9-nickase, which is a Cas9 variant with a mutated nuclease domain that introduces single-stranded instead of DSBs, with a base deaminase. Base editors are able to precisely catalyse base pair transition mutations without the requirement for HDR, making them suitable for correction of point mutations in neurons [27]. At present, there are two classes of base editor: cytosine base editor (CBE), which converts C^**.**^G to T^**.**^A, and adenine base editor (ABE), which converts A^**.**^T to G^**.**^C [27, 28]. Currently described base editors can reverse all possible transition mutations, which, according to current estimates, account for 25% of all human pathogenic point mutations [26, 29]. Importantly, more than 14,282 pathogenic missense and nonsense variants linked to genetic epilepsy have already been identified [30].

At present, there are no reports on the use of base editing in central nervous system (CNS) neurons [26]. However, its application to genetic epilepsy should be carefully considered. A recurrent point mutation in an epilepsy gene would be the optimal condition to be approached with base editing. The remarkably penetrant progressive myoclonic epilepsy *KCNC1* variant (c.959 G > A) is a prime example [31, 32]. Recurrent mutations have also been identified in *SCN1A* [33, 34] and *SCN8A* [35] although the incidence is not high. Dominant negative mutations, such as the *GABRG2* Q390X mutation [36, 37], can in principle be tackled with base editing, because other approaches such as gene supplementation are not suitable due to the dominant negative action of the mutant protein.

Recently Prime editing has been developed as another method of precisely installing point mutations and specific sequences into genes [38]. Although it has yet to be reported for neurons in vivo, this approach is able to rewrite, insert, and delete DNA sequences, without double-strand breaks or the need for a donor template, increasing the possibility of rescuing most mutations leading to genetic epilepsy. Indeed, it has been estimated that it could correct 89% of known genetic variants [29]. Notably, these technologies have the advantage of reduced genotoxicity, cellular stress responses, and the likelihood of large chromosomal rearrangements, compared to the Cas9 nuclease-based approaches, because they do not introduce DSBs.

## CRISPR-Mediated Gene Modulation for Genetic Epilepsies

CRISPR/Cas endonucleases permanently alter the genetic code, offering the potential to irreversibly cure disease, but also increasing the possibility of genotoxicity [39]. The specific and reversible modulation of gene expression, therefore, represents a desirable application of the CRISPR/Cas system. Gene modulation is achieved using an engineered dCas protein, which lacks endonuclease function, tethered to effector proteins that possess regulatory functions [21, 38, 40, 41]. dCas binding to a regulatory genomic locus results in the recruitment of endogenous cellular factors that promote or inhibit gene expression, strategies termed CRISPR activation (CRISPRa), and CRISPR interference (CRISPRi), respectively.

### CRISPR for the Treatment of Haploinsufficiency

Haploinsufficiency resulting from LOF mutations is the underlying molecular cause in the vast majority of cases for the ‘top ten’ most common paediatric epilepsy genes. Patients are heterozygous for such mutations [9] allowing for therapeutic intervention via upregulation of the WT allele using CRISPRa. The therapeutic utility of such an approach was recently demonstrated for Dravet syndrome, where CRISPRa was used to upregulate the expression of the WT *Scn1a* allele by ~ 50%, and improved the epileptic phenotype [42]. This ‘one-size-fits-all’ promoter-based strategy is advantageous in the context of monogenic epilepsy, as a significant proportion of mutations arise de novo.

Another strategy to manipulate gene expression is to modify the epigenome [43]. Epigenome editors have been developed that change chromatin structure via methylation and acetylation (reviewed in [43]). The development of epigenome editors has expanded the targeting range of CRISPR, as these tools are not restricted to targeting near to the transcription start site (TSS) [43, 44] and can activate enhancer elements that are more resistant to CRISPRa elements (dCas9-VP64) [45]. Considering that protein coding sequences form only 1 to 2% of the genome, with at least 8% of the genome consisting of known regulatory regions [46], these novel editors hold enormous therapeutic potential. Epigenome editors have already been applied to genetic epilepsy. For example, rescue of X-linked mutations in CDKL5 has been recently demonstrated using dCas9 fused to TET1, a demethylation factor. Demethylation of the CDKL5 promoter by dCas9-TET1 targeting was able to reactive the silenced WT X-allele in vitro [47].

### CRISPR for the Treatment of Gain-of-Function Mutations in Genetic Epilepsy

GOF mutations are also common in genetic epilepsy, for example in *SCN2A*, *SCN8A,* and *KCNT1* [48, 49], and the mechanistic target of rapamycin (mTOR) pathway [50]. Ion channel ‘GOF’ is most commonly used to refer to mutations that result in an increase in total current; however, it can also encompass a complex myriad of potential changes in channel kinetics, altering channel activation, inactivation, or deactivation [51], which can alter neuronal properties in ways that are not treatable by decreasing overall channel expression.

A general approach using CRISPRi or dCas9-methyltransferase fusions, directed to the promoter of these genes to decrease gene expression, could be considered in some cases. In support of this strategy, recent data has shown that antisense oligonucleotide targeting of *Scn8a* mRNA is able to rescue the epileptic phenotype in GOF genetic mouse models [52]. Moreover, focal cortical dysplasia, the commonest indication for epilepsy surgery in children [53], is caused in the vast majority of cases by somatic LOF mutations that lead to an overall GOF activity of the mTOR pathway [54]. Drug treatments [55] and antisense oligonucleotide therapies [56] targeting key components of the mTOR pathway have been shown efficient in improving the seizure phenotype in animal models and patients. A CRISPRi approach for these pathologies could be potentially implemented.

## Considerations on the Use of Gene Editing and Manipulation for Genetic Epilepsies

### The Importance of Gene Dosage in the Treatment of Genetic Epilepsies

Haploinsufficiency affects genes that display dosage sensitivity, whereby having 50% less of the protein product elicits disease. Dosage sensitivity also provides a model in which having 50% more of the gene product would also be deleterious [57]. This is perhaps well exemplified by neurodevelopmental disorders with epilepsy [12], where copy number variants (CNVs) account for > 14% of cases [58]. The dosage of *MECP2*, a transcriptional repressor, has been shown to be fundamental, as both LOF and gene duplication lead to distinct neurological diseases [59, 60]. Dosage sensitivity is also observed for the voltage-gated sodium channels *SCN1A*, *SCN2A*, and *SCN8A*, which show different disease phenotypes when mutations are LOF or GOF [48].

Traditional gene therapy strategies for haploinsufficiency involve gene replacement, whereby the WT gene is delivered to the cell using a viral vector. However, the dosage sensitivity of many epilepsy genes, namely ion channels, would imply that these disorders would not benefit from an overexpression-style gene replacement therapy. CRISPR approaches that modulate endogenous gene expression hold multiple advantages over gene replacement strategies, as they allow for the production of multiple splice isoforms and the cell-type specific control of gene expression [41]. Furthermore, CRISPR can circumvent difficulties regarding the delivery of coding sequences that are too large to be packaged into an adeno-associated vector (AAV)—the gene therapy vehicle of choice for CNS applications—such as *SCN1A*.

Restoring the transcriptional output of haploinsufficient genes to physiological levels may still prove challenging. However, the expanding CRISPR toolbox may allow for fine-tuning of gene expression. Various permutations of the CRISPRa platform, resulting from the fusion of dCas9 to different effector domains, upregulate genes by different orders of magnitude [41]. For example, dCas9-VP64 is one of the “weakest” transcriptional activators, often requiring multiple targeting sgRNA to achieve significant upregulation [61]. Second generation dCas9 activators, such as VP64-p65-Rta (VPR) and synergistic activation mediator (SAM), appear to be much stronger, often requiring only one targeting sgRNA due to increased effector recruitment by multiple fused activator elements [62, 63]. SunTag, which uses a protein scaffold to amplify the recruitment of effectors to the dCas9 bound locus, is the most potent activator to date and is even able to reactivate regions of heterochromatin [64]. Rational design of CRISPRa targeting is also likely to be important in tuning gene expression. For example, a CRISPRa strategy to treat Dravet syndrome only required one sgRNA to achieve roughly twofold increase at the mRNA level with dCas-VP64 [42], whereas a different approach using the same activator required 4 sgRNA to achieve therapeutic rescue [65]. The first study targeted just upstream of the transcription start site (TSS) of the proximal promoter of the *Scn1a* gene, whereas the latter targeted the distal promoter.

An alternative strategy to maintain physiological gene dosage is to use epigenome editors, for which there is burgeoning literature on their application in vivo [41]. Unlike CRISPRa/i agents, epigenome editors do not override promoter function and can recapitulate endogenous mechanisms of gene expression regulation, for example activity-dependence [44].

Most neuronal genes are under tight regulatory control, governed by a number of elements with different additive or negative contributions to the overall output of the promoter. Understanding the regulatory landscape of epilepsy genes will prove important in the future when designing such gene therapy strategies and may also facilitate cell type specific targeting, as it has been shown that genes that are not constitutively expressed are under greater regulatory control by enhancers, and tend to have more cell type specific expression patterns [66], such as *SCN1A* [67]. A systematic comparison of different CRISPR agents for the modulation of gene expression could be necessary to understand better their collective and individual clinical potential.

### Homeostatic Compensation and Window of Intervention for Genetic Epilepsies

The importance of dosage reflects the crucial property of ion channels and synaptic proteins in the homeostatic control of neuronal excitability and their tight regulation at the level of transcription and translation [68, 69]. This phenomenon forms the basis of the synergy between different key players in maintaining proper excitability set-points despite physiological and pathological changes in gene expression [70]. Such homeostatic control is integrated at both the single cell and network level [71, 72]. The expression of ion channels and synaptic proteins is therefore dynamic and closely coupled with network activity.

The activity-dependent expression of several genes is pivotal for the formation of healthy mature neural networks during development, a sensitive period for a large majority of the monogenic epilepsies [73]. Changes in ion flux, mediated by mutation of a single gene embedded in a homeostatic hub, could result in wider changes in gene and protein expression, contributing towards aberrant network activity and possibly the formation of an epileptic brain [74]. One example is seen in the identification of a co-expression network of 320 genes (M30) found to be commonly disrupted in several epilepsies of different aetiology, demonstrating that we should think of genetic epilepsy not as the singular effect of a gene mutation, but as the result of global changes in gene expression [75]. Importantly, ion channels implicated in genetic epilepsy are functionally interconnected. For example, the balance between Nav1.1 (haploinsufficiency leading to Dravet Syndrome) and Kv3.1 (dominant negative leading to PME and neurodevelopmental alterations) [76] is crucial in determining the fast-spiking nature of interneurons [77], with the loss of one of these components potentially severely altering the function of the other. This has been recently shown for LOF of Nav1.2, which paradoxically results in an increase in intrinsic excitability by preventing effective AP repolarisation by potassium channels [78]. Another example is the recent finding that *PRRT2* acts as negative modulator of Nav1.2/1.6, possibly explaining why patients with *PRRT2* mutations respond well to sodium channel blockers [79]. Furthermore, it was recently shown that antisense oligonucleotide treatment to decrease *Scn8a* (Nav1.6) expression was sufficient to rescue disease phenotypes in a Dravet Syndrome model of *Scn1a* haploinsufficiency [52]. These findings confirm the functional interconnection of different classes of protein in the control of neuronal excitability which, when mutated, lead to genetic epilepsies.

Furthermore, we have recently shown that upregulating *Kcna1* in an acquired epileptic network not only decreases neuronal excitability and seizure frequency, but can also restore the pathologically altered transcriptome and rescue cognitive comorbidities [80]. This finding underlines that gene regulation and network activity are tightly correlated, and illustrates how a small change in gene expression can result in more profound global effects [72].

The ability of gene editing to rescue the global transcriptomic effects of a single gene mutation remains to be seen. It is known that neurons and neuronal networks compensate over time for the loss of gene function [81–83]. It has been shown, for example, that there is a transient impairment of cortical fast-spiking PV interneurons in *Scn1a* haploinsufficient mice, with a normalisation of excitability by P35 [82]. However, at this stage, mice still experience seizures, raising the question—what is the underlying mechanism of seizure generation in this genetic epilepsy?

Important questions related to gene editing for epilepsy remain unanswered. What is the ideal window of intervention to correct a mutated gene? Is it enough to simply restore a physiological level of gene expression and can gene editing rescue developmental and cognitive comorbidities? Some studies have already shown that a window of intervention exists for the full rescue of phenotypes associated with genetic epilepsies. Some of these studies suggested the importance of early intervention, such as for Angelman Syndrome [84] and for *KCNQ2*/*KCNQ3*-associated epilepsy syndrome [85]. However, other studies suggested that also a later intervention during adulthood may be sufficient to rescue severe phenotypes, i.e. seizures and memory deficits, as seen for *SYNGAP1* haploinsufficiency [86]. Answering such questions for other genetic epilepsies would allow for more effective therapeutic interventions [87]. The continued development and characterization of CRISPR tools in relevant disease models can provide answers to these important questions.

### Limitations of CRISPR Mediated Gene Editing and Modulation for Epilepsy

Although CRISPR/Cas has advantages over classical gene therapy approaches, limitations involving CNS delivery, low editing efficiency, and off-target effects may hinder its rapid adoption into the clinic (reviewed elsewhere: [4, 88]). Other limitations include the difficulty of packaging some CRISPR tools (e.g. base and prime editors) into currently used AAV vectors due to limitations in the size of the genetic payload, and difficulties in the delivery of these tools to the human brain, both for safety (e.g. possible immunological responses to CRISPR proteins) and technical reasons (e.g. how we might bypass the blood–brain barrier and achieve a high transduction efficiency) [4, 12, 21, 88]. Novel tools and technologies are constantly emerging that aim to overcome these fundamental limitations for translation, however, suggesting that these challenges will be met in the near future.

With specific regard to epilepsy, low efficiency of CRISPR-mediated editing would result in genetic mosaicism, which could itself lead to aberrant network activity and maladaptive compensations. Certainly, mosaicism itself is pathogenic in certain epilepsies, such as X-linked mutations in *PCDH19* [89]. Mosaicism is also common for *CDKL5*, *SCN2A*, and *SCN1A* [90] and the exact mutational burden required to form an epileptic network is still not fully understood. Interestingly, a recent study addressed this question for *Scn8a* mutation. They showed that the presence of a GOF mutation in *Scn8a* n in > 16% of neurons was sufficient to reduce seizure threshold, whilst mutation in > 50% of neurons generated an epileptic network, suggesting that a high efficiency CRISPR therapy would be required to rescue this genetic epilepsy [91].

Despite these limitations, the use of CRISPR to correct and modulate gene expression in genetic epilepsy is a fundamental stepping-stone for better understanding and treating these devastating and life-threatening pathologies.

## Conclusion

**Fig. 1:**
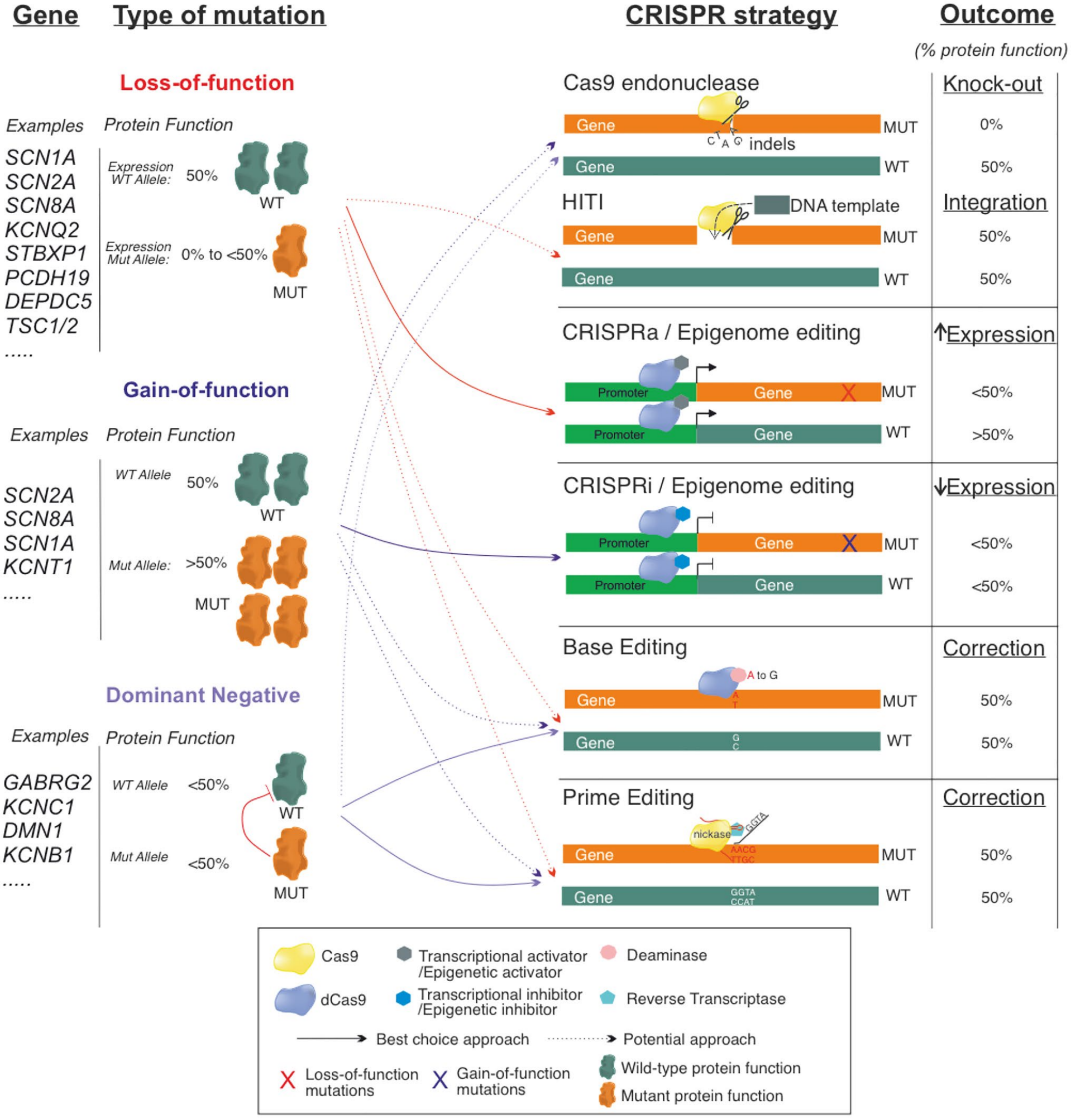
CRISPR/Cas-based strategies for genetic epilepsies. The impact of different types of epilepsy mutation is represented as a % of protein function, with each allele contributing 50% towards total protein function under normal physiological conditions. Reduced protein function, as a result of loss-of-function mutations or dominant negative repression of WT protein function, results in < 100% of protein functionality (i.e. the functional output is < 50% for one allele or for both alleles, respectively). For gain-of-function mutations, protein function is increased, which can be conceptualised as one allele contributing > 50% towards protein functionality. Gene editing/modulation strategies allow for the ‘normalisation’ of protein function to physiological levels by either increasing or decreasing the functional output of the WT or mutant allele. Abbreviations: Indels insertions or deletions, HITI homology-independent targeted integration, dCas9 catalytically deactivated Cas9, CRISPRa CRISPR activation, CRISPRi CRISPR interference, WT wild-type, MUT mutant

We now have a variety of CRISPR-based genetic tools that are able to potentially treat all the mutations that lead to genetic epilepsy (Fig. 1). A proper analysis of the underlying epileptogenic mechanisms, appropriate gene dosage, window of intervention, and potential molecular network alterations will be necessary to move towards the clinic. On the other hand, improvements in the delivery, efficiency and off-target effects of CRISPR tools must occur in parallel to hasten their translational applicability in the genetic epilepsies and other neurological diseases.

## Supplementary information

Below is the link to the electronic supplementary material.

Supplementary file1 (PDF 386 KB)

Supplementary file2 (PDF 1182 KB)
